# Pseudo-bilayer architecture enables high-performance organic solar cells with enhanced exciton diffusion length

**DOI:** 10.1038/s41467-020-20791-z

**Published:** 2021-01-20

**Authors:** Kui Jiang, Jie Zhang, Zhengxing Peng, Francis Lin, Shengfan Wu, Zhen Li, Yuzhong Chen, He Yan, Harald Ade, Zonglong Zhu, Alex K.-Y. Jen

**Affiliations:** 1 Department of Materials Science and Engineering, City University of Hong Kong, Tat Chee Avenue, 999077 Kowloon, Hong Kong; 2 Department of Chemistry, City University of Hong Kong, Tat Chee Avenue, 999077 Kowloon, Hong Kong; 3 Department of Physics and Organic and Carbon Electronics Laboratories (ORaCEL), North Carolina State University, Raleigh, NC 27695 USA; 4 Department of Chemistry and Energy Institute, The Hong Kong University of Science and Technology, Clear Water Bay, 999077 Kowloon, Hong Kong

**Keywords:** Solar cells, Solar cells

## Abstract

Solution-processed organic solar cells (OSCs) are a promising candidate for next-generation photovoltaic technologies. However, the short exciton diffusion length of the bulk heterojunction active layer in OSCs strongly hampers the full potential to be realized in these bulk heterojunction OSCs. Herein, we report high-performance OSCs with a pseudo-bilayer architecture, which possesses longer exciton diffusion length benefited from higher film crystallinity. This feature ensures the synergistic advantages of efficient exciton dissociation and charge transport in OSCs with pseudo-bilayer architecture, enabling a higher power conversion efficiency (17.42%) to be achieved compared to those with bulk heterojunction architecture (16.44%) due to higher short-circuit current density and fill factor. A certified efficiency of 16.31% is also achieved for the ternary OSC with a pseudo-bilayer active layer. Our results demonstrate the excellent potential for pseudo-bilayer architecture to be used for future OSC applications.

## Abstract (web summary)

The so-called pseudo-bilayer (PB) organic solar cell (OSC) device architecture can promote enhanced exciton dissociation and charge transport, leading to improved device performance. Here, the authors report high-efficiency OSCs that features a PB architecture and optimized ternary system.

## Introduction

Organic solar cells (OSCs) have been regarded as a promising new photovoltaic technology due to their advantages of low-cost solution processing, good flexibility, lightweight, and compatibility with various applications^1–3^. To achieve high performance in an OSC, it is critical to have proper film morphology in its photoactive layer in order to maintain a balance between exciton dissociation and charge transport. In the past few years, bulk heterojunction (BHJ) architecture has gained great success in OSC based on polymeric donors and fullerene-derived acceptors^4^, which circumvents the short exciton diffusion length in polymer donor materials (typically 5–10 nm)^5,6^ by providing an interpenetrating donor/acceptor (D/A) network for electron- and hole-transport^7^. This approach has successfully increased the short-circuit current density (*J*_SC_) in OSCs. Nevertheless, the BHJ OSCs based on fullerene-derivatives are also hampered by the inefficient charge transport in a complex D/A network. Until recently, significant efforts were devoted to designing novel donors and non-fullerene acceptors (NFAs)^8–13^ and device optimization^14–16^, for fullerene-free OSCs. The combination of new NFAs and donor materials reduce the energy loss and broaden the light absorption in BHJ OSCs, leading to a certified power conversion efficiency (PCE) of over 17%^17^. However, due to the short exciton diffusion length of BHJ active layer in OSCs, it is still necessary to maintain the complex D/A network, which compromises the charge transport in these BHJ OSCs.

It has been reported that larger domain size and higher domain purity will enhance the charge transport in OSCs^18^. Inspired by these findings, the pseudo-bilayer (PB) architecture, which is composed of three layers subsequently: a pure donor (or acceptor) layer at the bottom, a D/A mixture layer in between, and a pure acceptor (or donor) layer on top, has the potential to further alleviate the trade-off between exciton dissociation and charge collection^19,20^. Considering the special characteristics needed for PB architecture, only a few cases have been conducted so far.

Min et al. have fabricated the OSCs based on NFAs through layer-by-layer solution processing^21–23^. These OSCs possessed a PB architecture and exhibited better performance than the reference devices with conventional BHJ architecture. Success has also been achieved with sequential deposition in the FTAZ:IT-M binary and FTAZ:DPP-3T:PEBM ternary blends. Improved molecular packing and domain purity in the layered device also resulted in higher performance than those of the reference BHJ devices^24,25^. Although the highest PCE achieved so far for these PB-based OSCs is only 16.5%^23^, these works demonstrate the great potential for further improvements in NFA-based OSCs, which warrants a deeper understanding of the reasons for high performance achieved.

Herein, we report high-efficiency OSCs with a PB architecture that outperform conventional BHJ devices. The results are based on a ternary system containing the donor poly[(2,6-(4,8-bis(5-(2-ethylhexyl-3-fluoro)thiophen-2-yl)-benzo[1,2-*b*:4,5-*b*′]dithiophene))-*alt*-(5,5-(1′,3′-di-2-thienyl-5′,7′-bis(2-ethylhexyl)benzo-[1′,2′-*c*:4′,5′-*c*′]dithiophene-4,8-dione))] (PM6)^26^, and the acceptors 2,10-bis(2-methylene-(3-(1,1-dicyanomethylene)-5,6-difluoroindanone))-12, 13-bis(3-ethylheptyl)-3, 9-diundecyl-dithieno-[2″, 3″:4′, 5′] thieno[2′, 3′:4, 5] pyrrolo[3, 2-*e*:2′,3′-*g*] [2, 1, 3] benzothiadiazole (N3)^27^ and [6,6]-phenyl-C_71_-butyric acid methyl ester (PC_71_BM).

The grazing-incidence wide-angle X-ray scattering (GIWAXS) measurements revealed that both the crystallinity and domain purity in PB films are higher than those in BHJ films. The steady-state PL spectra showed the vertical gradient distribution of donor and acceptor in PB films, where the higher concentration of donor/acceptor can be identified near cathode/anode, respectively. The carrier mobility in the PB film was also enhanced, implying improved charge transport and collection. Furthermore, the exciton diffusion length increased from 7.53 nm in the BHJ architecture to 17.07 nm in the PB architecture, which should be attributed to the longer coherence length (CL) of the aggregates in the PB films benefited the exciton dissociation in the PB OSCs. These lead to high *J*_SC_ in the PB OSCs without sacrificing the fill factor (FF). The champion PB OSC achieved an impressive PCE of 17.42%, which is significantly higher than that of the BHJ OSC (16.44%).

The similar PB concept was applied to another low bandgap NFA, 2,10-bis(2-methylene-(3-(1,1-dicyanomethylene)-5,6-difluoroindanone))-12,13-bis(2-ethylhexyl)-3,9-bis-(undecyloxy)-dithieno[2″,3″:4′,5′]thieno[2′,3′:4,5]pyrrolo[3,2-*e*:2′,3′-*g*][2,1,3]benzothiadiazole (Y6-O). The PB devices based on PM6:Y6-O:PC_71_BM also exhibited a high PCE of 17.27% and achieved a certified PCE of 16.31%, which is the highest performance reported so far for PB OSCs. These results not only show the correlation between active layer architecture, charge transport, and performance in high-efficiency PB OSCs, but also demonstrate its potential in further improving OSC performance.

## Results

### Morphological characterizations of films

**Fig. 1:**
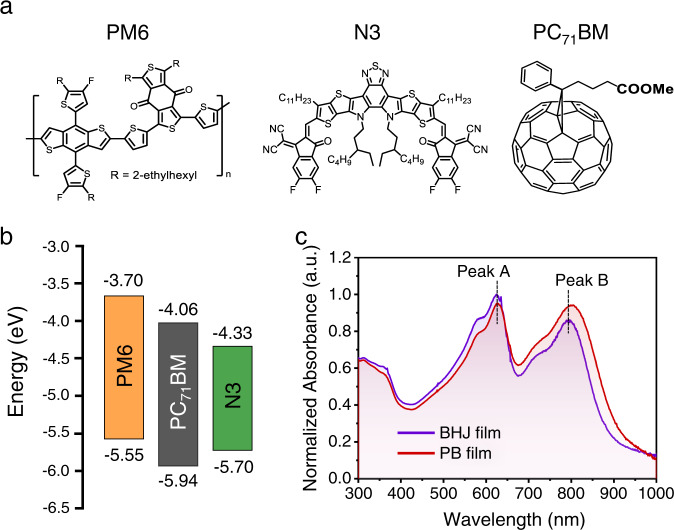
Molecular structures of donor/acceptors and absorbance of films. **a** Chemical structures and **b** energy levels of PM6, N3, and PC_71_BM. **c** UV–Vis absorption spectra of PM6:N3:PC_71_BM blend film with BHJ or PB architectures.

The chemical structures, energy levels, and UV–Vis absorption spectra of donor (PM6) and acceptors (N3, PC_71_BM) used in this work are illustrated in Fig. 1a, b and Supplementary Fig. 1, respectively. The UV–Vis absorption spectra of films with BHJ and PB architectures implies that PM6 and N3 dominated the film absorption, where Peak A and Peak B represent PM6 and N3, respectively (Fig. 1c). The detailed absorption profiles show that the BHJ film possessed better light-harvesting from ~300 to 645 nm, while the PB film exhibited stronger absorption in the range between ~645 and 1000 nm.

**Fig. 2:**
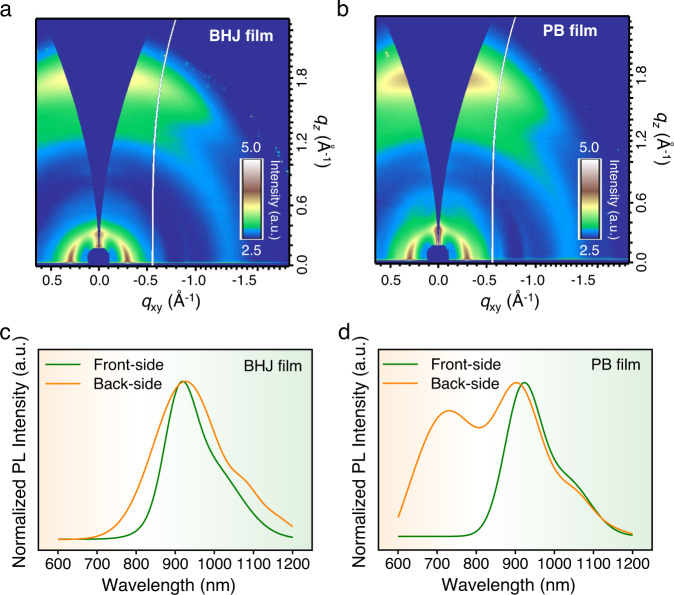
Morphological characterization for films with BHJ and PB architectures. **a, b** 2D GIWAXS patterns of PM6:N3:PC_71_BM blend film with **a** BHJ and **b** PB architectures. **c**, **d** The PL spectra of the films with **c** BHJ and **d** PB architectures, which were excited from the front- and back-sides.

The micro-morphological structures of the films based on PM6, N3, and PC_71_BM were carefully studied by GIWAXS measurements^28^. The GIWAXS patterns and profiles of neat films of PM6, N3, and PC_71_BM are shown in Supplementary Fig. 2. Both PM6 and N3 showed preferential face-on orientation relative to substrate as evidenced by the (100) peak at *q* ~ 0.3 Å^−1^ in the in-plane (IP) direction with the strong (010) peak (*q* ~ 1.72 Å^−1^ for PM6 and *q* ~ 1.83 Å^−1^ for N3) in the out-of-plane (OoP) direction. Comparatively, the OoP and IP GIWAXS profiles of PC_71_BM showed similar peaks at *q* = 0.65 Å^−1^, *q* = 1.35 Å^−1^, and *q* = 1.90 Å^−1^, indicating no obvious molecular orientation. Based on these results, the morphological structures of PM6:N3:PC_71_BM blend films were studied from the GIWAXS patterns and profiles. The signal intensity of the PB film, especially in the OoP direction, is higher than that of the BHJ film (Fig. 2a, b), implying the higher degree of molecular packing in the PB film.

The parameters of GIWAXS 1D line-cut profiles in (Supplementary Fig. 3) are summarized in Supplementary Table 1, where the (010) peak at *q* = 1.71–1.73 Å^−1^ corresponds to PM6, and the peak at *q* = 1.77–1.79 Å^−1^ is from N3. The noticeable scattering intensities of the PC_71_BM peak at *q* ~ 0.65 Å^−1^, is indicative of fullerene aggregation outside mixed domains^29,30^, which both increased for the PB film. These aggregated PC_71_BM domains could provide better electron-transporting pathways and thus improve the charge collection in devices. In the PB film, the normalized intensity (normalized by scattering volume) of π–π stacking peak in OoP and the corresponding coherence length are improved slightly (Supplementary Table 1), which would contribute to an enhanced OoP charge transport in film^31^. It is noteworthy that the stronger stacking can be correlated to a more narrow bandgap of small molecules in thin film, leading to a redshift of optical absorption^32^. As shown in Fig. 1c, the Peak A of BHJ and PB films both locate at about 625.5 nm, while the Peak B of PB film exhibits a redshift of 10.2 nm compared to that of BHJ film. Therefore, we speculate that the different UV–Vis absorption profiles of the BHJ and PB films, as described above, may be ascribed to the variation of stacking patterns in the films.

The resonant soft X-ray scattering (R-SoXS) measurements were carried out to explore the properties concerning domains spacing statistics in the films (Supplementary Fig. 4 and Supplementary Table 2)^33–35^. Both the BHJ and PB film show bimodal spacing distribution with a low-*q* peak at ~0.1 nm^−1^, and a high-*q* peak shoulder at ~0.3 nm^−1^. The overall root-mean-square variation of the composition of PB film (1.00) is higher than that of BHJ film (0.94). Since the scattering contrast is dominated by the contrast differences to the PC_71_BM (Supplementary Fig. 5), this indicates higher average domain purity for the PC_71_BM distribution in the PB film. Moreover, the PB film exhibited a larger long period for the low-*q* peak and average domain size, suggesting a more pronounced phase separation in this film. This result is consistent with the higher intensity of the fullerene GIWAXS peak at *q* ~ 0.65 Å^−1^, which implies the suitable vertical phase separation in the PB film due to the increased fullerene aggregation outside mixed domains. The phase separation and pure domains reduce recombination^36^ and benefit the charge collection in devices based on the PB films^31^. These observations are similar to prior PB work^24^.

Furthermore, the macro-morphology of films was investigated indirectly via photoluminescence (PL) measurements with an excitation wavelength of 550 nm. The PL spectra measured with the near-infrared (NIR) detector were fitted by the multi-peak fitting method for the following PL analysis (Supplementary Fig. 6), owing to the low signal-to-noise ratio (SNR). It is worth to point out that PC_71_BM would not quench the PL of N3 domains due to the type-I energy alignment (Fig. 1b). As shown in (Supplementary Fig. 7) the PL signals of neat PM6 and N3:PC_71_BM films were collected in the range between 600 and 1200 nm (peak located at 750 nm) and 800–1200 nm (peak located at 928 nm), respectively, implying lower energy of singlet exciton in N3. Although the absorption coefficient of PM6, N3, and PC_71_BM indicated that the excitation light of 550 nm would be mainly absorbed by PM6 domains in the blend film (Supplementary Fig. 1), the PL spectrum for a blend film would be dominated by the N3 domains and the charge-transfer (CT) states at the interface between PM6 and N3, which is due to the energy transfer^37–39^, unless the excited area is composed of pure PM6 domains and distant from acceptor domains^40–42^.

Given the collected PL signals were mainly contributed by the excited side, the PL measurements were conducted by exciting the front- or back-side of the films to deduce the vertical distribution of PM6 in the films (Supplementary Fig. 8). As shown in Fig. 2c, the PL spectra collected from both the front- and back-sides of BHJ film were dominated by the N3 domains. This means that the donor domains blended well with the acceptor domains at either front- or back-side of BHJ film. In contrast, the PL spectra of PB film is significantly dependent on the directionality of excitation (Fig. 2d). The PL signals originated from PM6 domains (from 600 to 800 nm) were observed with the back-side excitation, while it disappeared under front-side excitation. According to the PL peaks of neat PM6 and N3:PC_71_BM films (Supplementary Fig. 7), these results demonstrate that the front- and back-sides of PB film were dominated by the N3:PC_71_BM and pure PM6 domains, respectively, while the blended PM6:acceptor (N3, PC_71_BM or N3:PC_71_BM) domains exist throughout the whole BHJ film. Moreover, we conducted the time-of-flight secondary ion mass spectrometry (ToF-SIMS) measurements to verify the vertical phase morphology of the film with BHJ and PB structures (Supplementary Fig. 9). The -CN^−^ group was used to track the N3 small molecule. The depth profile of the BHJ film was relatively flat throughout the ToF-SIMS measurement. However, the characteristic mass fragments of the PB film showed the obviously enriched N3 at the top region of the film, while the N3 concentration decreased continuously to form a plateau in the middle region of the film. This result demonstrated that the PB structure possessed the neat N3:PC_71_BM layer, mixed layer and neat PM6 layer from bottom to top, which was consistent with the PL results in Fig. 2c, d. The vertical morphology of films with BHJ and PB architectures is illustrated in (Supplementary Fig. 10).

### Device performance

**Fig. 3:**
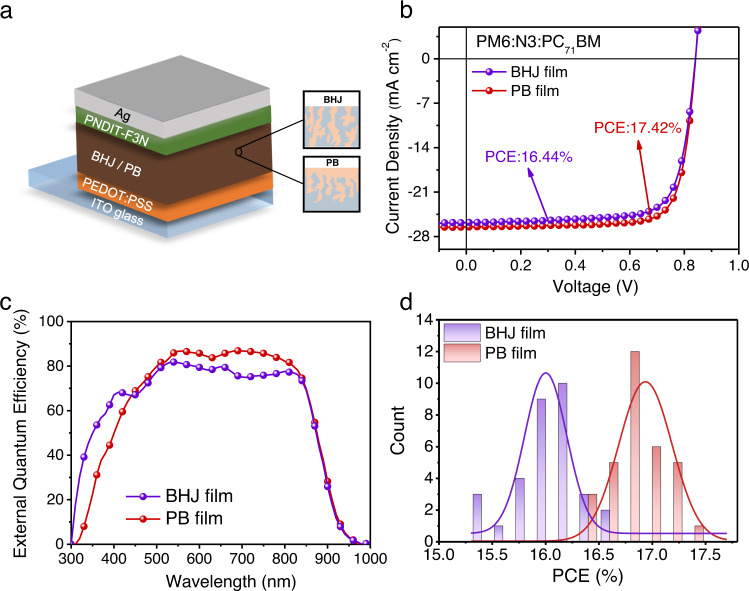
Device structure and performance. **a** Schematic of OSC structure. **b** *J*–*V* curves of the OSCs with BHJ and PB architectures under 1 sun illumination. **c** EQE spectra of the BHJ and PB OSCs. **d** PCE histograms of BHJ and PB OSCs.

To inspect the performance of the devices with the BHJ and PB architectures, the OSCs were fabricated based on the spin-coating method with a device configuration of indium tin oxide (ITO) glass/poly(3,4-ethylenedioxythiophene): polystyrene sulfonate (PEDOT:PSS)/PM6:N3:PC_71_BM /poly[[2,7-bis(2-ethylhexyl)-1,2,3,6,7,8-hexahydro-1,3,6,8-tetraoxobenzo[lmn][3,8] phenanthroline-4,9-diyl]-2,5-thiophenediyl[9,9-bis[3-(dimethylamino)propyl]-9H-fluorene-2,7-diyl]-2,5-thiophenediyl] (PNDIT-F3N)^43^/Ag (Fig. 3a). The current density–voltage (*J*–*V*) curves of the champion OSCs based on BHJ film and PB film are presented in Fig. 3b. The BHJ OSC possessed a *V*_OC_ of 0.840 V, a *J*_SC_ of 25.69 mA cm^−2^, an FF of 0.762, and a PCE of 16.44%. As expected, the PCE of PB OSC was improved to 17.42% with a *V*_OC_ of 0.841 V, a *J*_SC_ of 26.49 mA cm^−2^, and an FF of 0.782 (Table 1), which is among the highest PCE in the reported OSCs. The *J*_sc_ obtained from the *J*–*V* curves matched well with the integrated value extracted from the external quantum efficiency (EQE) spectra (Fig. 3c and Table 1), indicating the good reliability of the results. The PCE histograms (obtained from 30 devices) in Fig. 3d show the enhanced performance and the good reproducibility of the PB OSCs. The box-plots of the photovoltaic parameters illustrated that the improved PCE was mainly due to the *J*_SC_ and FF enhancement (Supplementary Fig. 11 and Supplementary Table 3).

**Table 1:** The performance parameters of the champion devices of BHJ and PB architectures.

Architecture	*V*_OC_ (V)	*J*_SC_ (mA cm^−2^)	FF (%)	PCE (%)
BHJ	0.840	25.69 (24.98)^a^	76.2	16.44
PB	0.841	26.49 (25.79)	78.2	17.42

^a^EQE values.

### Performance analysis

It has been reported that the charge transport and collection affect the FF in solar cells^44^, thus the electrical properties of BHJ and PB OSCs were carefully studied. The space-charge-limited-current (SCLC) method was first employed to measure the charge carrier mobility in devices. As shown in Supplementary Fig. 12 and Supplementary Table 4, the mobilities of the electron (*μ*_e_) and hole (*μ*_e_) in the PB OSC were 23.91% and 18.33% higher than those in the BHJ OSC, respectively, proving better charge transport in the PB film. The higher carrier mobilities in PB film would be ascribed to the higher out-of-plane crystallinity (Supplementary Fig. 3)^45,46^. Moreover, the value of *μ*_h_/*μ*_e_ ratio in the PB OSC (1.24) is closer to unity than that in the BHJ OSC (1.30), implying a better-balanced electron–hole transport that would benefit charge collection^27,47^. The light intensity (*I*) dependent *V*_OC_ and *J*_SC_ were collected to investigate the carrier recombination behaviors in devices. The relation between *J*_SC_ and *I* is described as *J*_SC_ ∝ *I*^*α*^, and the *α* values extracted from logarithmic *J*_SC_–*I* plots were 0.981 and 0.994 for the BHJ and PB OSCs, respectively, implying that the bimolecular recombination in PB OSC was suppressed (Supplementary Fig. 13a)^48^.

In addition, the slopes of the semi-logarithmic open-circuit voltage–light intensity (*V*_OC_–*I*) plots were 1.11 *k*_B_*T*/*e* and 1.05 *k*_B_*T*/*e* for the BHJ and PB OSCs, respectively, where *k*_B_ is the Boltzmann constant, *T* is the absolute temperature and *e* is the elementary charge (Supplementary Fig. 13b). The smaller slope manifests the suppressed trap-assisted recombination in the pseudo-bilayer OSC^49^, which could be attributed to the suppressed carrier recombination or the faster charge extraction. Given the similar transient photovoltage (TPV) results of the BHJ (0.37 μs) and PB (0.38 μs) OSCs, the transient photocurrent (TPC) results (Supplementary Fig. 14) indicated the shorter carrier extraction lifetime in PB OSC (0.94 μs) compared to that in BHJ OSC (1.12 μs), implying the improved charge collection in the PB OSC. These results revealed that the morphology in PB film indeed enhanced charge transport and collection in the corresponding OSCs.

Moreover, the maximum FF (FF_max_) of a solar cell can be calculated with the equations listed below^50^:

1 $${\mathrm{FF}}_{\mathrm{max}} = \frac{{\nu _{\mathrm{OC}} - \ln \left( {\nu _{\mathrm{OC}} + 0.72} \right)}}{{\nu _{\mathrm{OC}} + 1}}$$

2 $$\nu _{\mathrm{OC}} = \frac{{V_{\mathrm{OC}}}}{{nk_{\mathrm{B}}T/e}}$$

where *n* is extracted from the slope of *nk*_B_*T*/*e* in the semi-logarithmic *V*_OC_–*I* plot. The FF_max_ is the maximum FF of a solar cell when there is no charge transport loss inside. Therefore, the FF loss can be attributed by two components: non-radiative loss and charge transport loss. As shown in Supplementary Fig. 15, the non-radiative losses were similar in the BHJ and PB OSCs, however, the charge transport loss was obviously reduced in the PB OSC. These results revealed that the charge transport and collection in the PB OSC were indeed enhanced, leading to higher FF.

Furthermore, the effects of film morphology on the *J*_SC_ enhancements were explored. In general, better D/A blending leads to a smaller average domain size, which would benefit the exciton dissociation at the D/A interface due to the shorter exciton diffusion length. However, charge transport could be hindered by the longer transport path induced by a more complex donor and acceptor network. Therefore, the average domain size in the optimized BHJ OSCs is usually controlled at ~20 nm to obtain the proper balance between *J*_SC_ and FF, which are affected by exciton dissociation and charge collection, respectively.

Unexpectedly, although the average domain size in PB film (38.0 nm) is larger than that in BHJ film (26.5 nm), the *J*_SC_ in the OSCs based on PB films are even higher than those based on BHJ films, indicating the efficient exciton dissociation in PB OSCs. This is consistent with prior observations in PB devices^24^. To explore the underlying mechanism of this phenomenon, time-resolved PL (TRPL) measurements were conducted to study the dynamic exciton behavior in the films. Due to the limitation on the SNR of our NIR detector in detecting the weak NIR signals of blend films in TRPL measurements, the UV–Vis detector with better SNR was used for all TRPL experiments. The PL signals at 680 and 840 nm in blend films were designated for studying the exciton behaviors in PM6 and N3 domains, respectively (more details in Supplementary Note 1).

The PL decay plots of blend and neat films were shown in Supplementary Fig. 16. The BHJ and PB films both exhibited faster PL decay than the neat films at 680 and 840 nm, while the PL decay in PB film was slower than that of BHJ film. It is noteworthy that the PL decay at 840 nm is not impacted by the excitation power density (Supplementary Fig. 17), which indicates that the PL signals at 840 nm are originated from the geminate recombination, such as the singlet exciton in N3 and charge-transfer emission (CTE) at D/A interface because the photo energy of CTE should be lower or closed to the energy of singlet exciton in N3 (Supplementary Fig. 18). The lifetimes of PL decay in the blend and neat films were obtained by fitting to biexponential function and listed in Supplementary Table 5. In PB film, the lifetime of PL decay recorded at 680 and 840 nm were both longer than those in BHJ film, implying longer exciton lifetime in PM6 and N3 domains.

In general, the exciton dissociation has occurred at the D/A interface in OSCs, thus the exciton diffusion length (*L*_D_) is important for exciton dissociation because the excitons generated in the neat domains need to reach the D/A interface before being quenched. To reveal the mean of exciton lifetime for the exciton dissociation in this work, the *L*_D_ was estimated with the PL quenching method established by Markov et al. (more details in Supplementary Note 2)^51^. The quenching efficiency (*Q*) and exciton diffusion length for the BHJ and PB films are summarized in Supplementary Table 6.

**Fig. 4:**
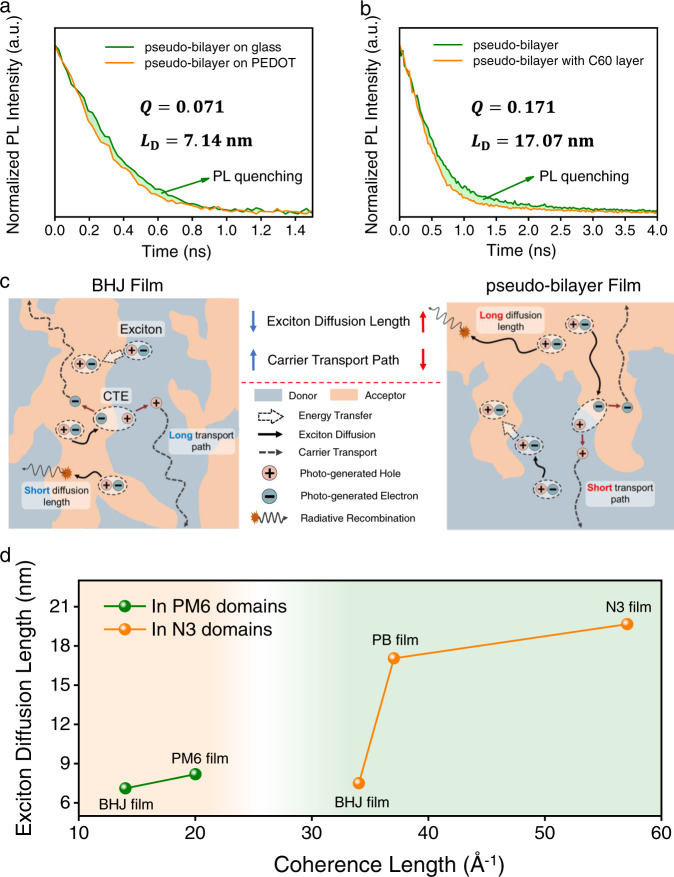
Performance analysis of OSCs. **a**, **b** PL quenching for the PB film prepared on **a** PEDOT layer and coated with **b** C_60_ layer, respectively. **c** Schematic of the behaviors of exciton and charge carriers in the BHJ and PB film. **d** Exciton diffusion length plotted as a function of CL in the domains.

When the films were prepared on PEDOT:PSS layer, the PL decay at 680 nm changed little for BHJ films (Supplementary Fig. 19a). This fast PL decay in BHJ films may be ascribed to the efficient exciton dissociation at the D/A interface and energy transfer from PM6 domains to N3 domains, which are due to the small domain size induced by the sufficient phase blending. However, a faster PL decay at 680 nm is observed for the PB film (Fig. 4a), resulting in an exciton diffusion length of 7.14 nm in PM6 domains for PB films. This short exciton diffusion length could be one of the reasons for the smaller EQE of PB devices in the range between 300 and 440 nm. Furthermore, as shown in Supplementary Table 6, the films with BHJ and PB structures showed similar thickness, which were 94 and 99 nm, respectively. It is noteworthy that the film with BHJ structure showed a higher absorption coefficient in the range between 300 and 440 nm (Supplementary Fig. 20), implying that more light was absorbed by the BHJ film in this wavelength range. Moreover, Supplementary Fig. 1 showed that the light in the range between 300 and 440 nm was mainly absorbed by the PC_71_BM and PM6 domains. For the film with PB structure, both the PL results (Fig. 2c, d) and ToF-SIMS results (Supplementary Fig. 9) indicate that there is a neat PM6 layer exist in the PB film, implying the PM6/PC_71_BM interfacial areas were smaller than those in the BHJ film. In addition, the average domain size in the PB film was larger than that in the BHJ film (Supplementary Table 2). These features mean that there are less dissociation but more quenching for excitons generated between 300 and 440 nm in the PB film, due to less interfacial area, shorter exciton diffusion length in PM6 domains (Supplementary Table 6), and larger domain size. Therefore, less photocurrents are generated in the range between 300 and 440 nm for the PB OSC than those for the BHJ OSC, due to inefficient exciton dissociation, resulting in lower EQE in the PB OSC.

For the films coated with a C60 layer, both the BHJ and PB films exhibit faster PL decay at 840 nm (Fig. 4b and Supplementary Fig. 19b). The exciton diffusion lengths are calculated to be 7.53 and 17.07 nm for the BHJ and PB films, respectively. The longer exciton diffusion length in PB film explains the efficient exciton dissociation with a larger average domain size (38 nm), owing to the ability of excitons to reach the D/A interface across the large acceptor domains before being quenched. The schematic of the behaviors of excitons and charge carriers in the BHJ and PB film is shown in Fig. 4c. Benefited by the longer exciton diffusion length and shorter carrier transporting path, both exciton dissociation and charge collection are simultaneously enhanced in the devices with PB architecture.

It has been reported that exciton diffusion length increases with the crystalline order in the organic semiconductors^52–56^. Therefore, the exciton diffusion length was plotted as a function of CL (Fig. 4d). This result implies that the longer exciton diffusion length may be benefited by the longer CL in the films, which is consistent with the higher crystallinity as shown in the GIWAXS results (Fig. 2a, b). Comparing the different film processing, the longer CL in the PB film could be ascribed to the sequential deposition (SD) processing method, which mitigates the influence of the acceptor (or donor) on the molecular stacking in the donor (or acceptor) domains during film preparation. A piece of evidence for this inference is that the normalized intensity of π–π stacking in OoP and the CL of these π–π stacking for PM6 and N3 in neat films are both higher than those in the blend films. Based on the features mentioned above, the PM6:N3:PC_71_BM films with PB architecture would enhance charge transport by stronger phase separation without sacrificing the overall ability of exciton dissociation in the PB OSCs, resulting in higher FF and *J*_SC_ observed, which is consistent with the previous study^57^.

### General applicability of SD processing strategy

In order to test the general applicability of using this SD processing method on other ternary OSCs, the device based on PM6:Y6-O:PC_71_BM was also fabricated with the same configuration. The BHJ OSC showed a *V*_OC_ of 0.900 V, a *J*_SC_ of 23.30 mA cm^−2^, an FF of 0.770, and a PCE of 16.14%, while the PCE of pseudo-bilayer OSC improved to be 17.27% due to a higher *J*_SC_ of 24.30 mA cm^−2^ and FF of 0.790 (Supplementary Fig. 21 and Supplementary Table 7). This showed that the performance of pseudo-bilayer OSC based on PM6:Y6-O:PC_71_BM could also be benefited by the enhanced charge transport and collection. In addition, the EQE of pseudo-bilayer OSC is higher than that of BHJ OSC from 300 nm to 380 nm, while it is lower from 380 nm to 900 nm. This phenomenon is similar to that observed for OSCs based on PM6:N3:PC_71_BM. More importantly, the PCE of the encapsulated pseudo-bilayer OSC was certified to be 16.31% by an independent accredited institute (the National Renewable Energy Laboratory, Colorado, USA) under the stress-test certification protocol at maximum power point (MPP), which is closed to the PCE measured in the lab (Supplementary Fig. 22).

## Discussion

In this work, the PB film based on PM6:N3:PC_71_BM was fabricated through the SD processing method. The results from GIWAXS showed that the OoP π-π stacking and crystallinity for both PM6 and N3 were enhanced in PB film. The R-SoXS results implied that the domains in PB film possess higher overall root-mean-square composition variation and is more phase-separated. Moreover, the steady-state PL spectra showed that the PM6:N3:PC_71_BM blended domains exist at the front- and back-sides of BHJ film, while the front- and back-sides of PB film were dominated by N3:PC_71_BM and PM6 domains, respectively. These morphological modifications improve the charge transport and collection in the PB OSCs, which is validated by a series of studies about the electrical properties of devices. Furthermore, the longer exciton diffusion lengths in the films are estimated with the TRPL measurements. The longer exciton diffusion length in the PB film benefited the efficient exciton dissociation in the films with larger domain sizes. Due to these advantages, the PCE of pseudo-bilayer OSCs increased from 16.44 to 17.42%, which is among the highest PCE of the reported OSCs. Furthermore, this PB architecture could also be applied to the devices composed of other Y6-derivative, PM6:Y6-O:PC_71_BM. The highest PCE of pseudo-bilayer OSCs based on PM6:Y6-O:PC_71_BM achieved 17.27% and with a certified value of 16.31% by NREL. Our results provide important insight for achieving high-performance NFA-based OSCs with PB architecture.

## Methods

### Materials

Donor polymer PM6 (*M*_n_: 24.2 kDa; *M*_w_: 88.0 kDa; PDI: 3.361) was provided by Solarmer Materials Inc. PC_71_BM was purchased from Sigma-Aldrich. N3 and Y6-O were synthesized according to the procedures in the literatures^27,58^.

### Device fabrication and characterization

All solar cells were fabricated in a conventional device configuration of ITO/PEDOT:PSS/active layers/PNDIT-F3N/Ag. The ITO glasses were cleaned with detergent and then sonicated with deionized water, acetone, and isopropanol sequentially. Then, the ITO glasses were dried overnight in an oven at 100 °C. The ITO substrates were treated with UV-Ozone for 30 min before using. The PEDOT:PSS (Heraeus Clevios P VP AI 4083) solution was spin-coated onto the treated ITO substrates with a speed of 5000 rpm for 30 s, and then dried at 120 °C for 15 min in air and were moved into an N_2_-filled glovebox. The PM6:SMA:PC_71_BM blends (1:0.96:0.24 weight ratio) were dissolved in chloroform with the donor concentration of 8 mg mL^−1^ and stirred at 55 °C for 2 h. 0.5% CN was added into the chloroform solution 30 min before the active layer was formed by spin coating at 1800–2800 rpm for 39 s followed by thermal annealing at 90 °C for 5 min. For the SD solution processing method, PM6 was dissolved in chloroform with the concentration of 8 mg mL^−1^, SMA:PC_71_BM blends were dissolved in chloroform (total concentration of blend solutions were 10 mg mL^−1^) with the addition of 0.5% CN, and stirred for 30 min on a hotplate at 55 °C. PM6 solution was spin-cast at 1800–2500 rpm for 39 s on the top of PEDOT:PSS layer and then the SMA:PC_71_BM blend solution was spin-cast on the top of PM6 film at 2000–2800 rpm for 30 s followed by a thermal annealing step at 90 °C for 5 min. The optimal thicknesses of the PM6 layer and PB film are about 50 and 99 nm, respectively. The thicknesses of the film were measured by a Bruker Dektak XT stylus profilometer. We estimate the thickness of the NFA layer by subtracting the PM6 layer thickness by the PB film thickness, which is about 49 nm. A thin PNDIT-F3N (0.5–1 mg mL^−1^ in methanol) layer was coated on the active layer. Later, Ag (~ 220 nm) was thermally evaporated at a vacuum level under 5 × 10^−5^ Pa through a shadow mask at rates between 2.5 and 5 Å s^−1^. The encapsulated device efficiencies were measured by a Keithley 2400 Source Meter in the air under AM 1.5 G (100 mW cm^−2^) using a Newport solar simulator. The light intensity was calibrated using a standard Si diode (with KG5 filter, purchased from PV Measurement) to bring spectral mismatch to unity. Typical cells have a device area of 5.9 mm^2^, defined by a metal mask with an aperture aligned with the device area. EQEs were measured using an Enlitech QE-S EQE system equipped with a standard Si diode. Monochromatic light was generated from a Newport 300 W lamp source. ToF-SIMS measurement was performed using a TOF-SIMS V instrument (ION-TOF GmbH, Cameca IMS 4F), where a 3 keV Cs^+^ ion beam was used for erosion and a 25 keV Bi^+^ pulsed primary ion beam was used for the analysis. The area of analysis was 48 × 48 μm^2^ while the sputtering area was 300 × 300 μm^2^.

### SCLC measurements

The hole-only and electron-only devices were fabricated by employing the following device structure: ITO/MoO_3_/blend film/MoO_3_/Al for holes and ITO/ZnO/blend film/PNDIT-F3N/Al for electrons. For the hole-only devices, MoO_3_ (~15 nm) was thermally evaporated onto the treated ITO substrates under a vacuum level of 5 × 10^−5^ Pa at rates between 2.5 and 5 Å/s. The photoactive layers were fabricated using the same conditions of solar cell devices. Later, MoO_3_ (~8 nm) and Al (~100 nm) was thermally evaporated at a vacuum level under 5 × 10^−5^ Pa through a shadow mask at rates between 2.5 and 5 Å/s. Electron-only devices were fabricated via the same methods used for solar cell devices just replaced the PEDOT:PSS with sol-gel ZnO. The thicknesses of the film were measured by a Bruker Dektak XT stylus profilometer. The mobilities were obtained by taking current–voltage curves and fitting the results to the equation listed below:

$$J = \frac{{9\varepsilon _0\varepsilon _{\mathrm{r}}\mu V^2}}{{8L^3}}$$

where *J* is the current density, *ε*_0_ is the vacuum permittivity, *ε*_r_ is the relative dielectric constant, *μ* is the mobility, *V* is the voltage, and *L* is the film thickness.

### Transient photocurrent (TPC) and transient photovoltage (TPV) measurements

The devices were mounted on a conductive clip and under steady-state illumination from a focused Quartz Tungsten-Halogen Lamp light source. The analyses were performed with a background response similar to open-circuit voltage. A digital oscilloscope acquired the TPV signal at the open-circuit condition. An optical perturbation is applied to the device with a 1 kHz femtosecond pulse laser under 640 nm excitation. TPC signal was measured under approximately short-circuit condition by applying a 100 Ω resistor.

### GIWAXS characterization

GIWAXS measurements were performed at beamline 7.3.3 at the Advanced Light Source. Samples were prepared on Si substrates using identical blend solutions and conditions as those used in OPV fabrication. The 10 keV X-ray beam was incident at a grazing angle of 0.12°–0.15°, which maximized the scattering intensity from the samples. The scattered X-rays were detected using a Dectris Pilatus 1-M photon-counting detector. Samples were prepared on Si substrates. All measurements were conducted under a helium atmosphere to reduce air scattering. In-plane and out-of-plane sector averages were calculated using the Nika software package. The uncertainty for the peak fitting of the GIWAXS data is 0.3 A. The coherence length was calculated using the Scherrer equation: CL = 2π*K*/Δ*q*, Where Δ*q* is the full-width at half-maximum of the peak and *K* is a shape factor (0.94 was used here).

### R-SoXs characterization

R-SoXS transmission measurements were performed at beamline 11.0.1.2 at the Advanced Light Source. Samples for R-SoXS measurement were prepared on a PSS modified Si substrate under the same conditions as those used for device fabrication, and then transferred by floating in the water to a 1.5 mm × 1.5 mm, 100 nm thick Si_3_N_4_ membrane supported by a 5 mm × 5 mm, 200-µm-thick Si frame (Norcada Inc.). Two-dimensional scattering patterns were collected on an in-vacuum CCD camera. The composition variation (related to the relative domain purity) over the length scales probed can be extracted by integrating scattering profiles to yield the integrated scattering intensity (ISI). The purer the average domains are, the higher the ISI. Owing to a lack of absolute flux normalization, the absolute composition cannot be obtained only by R-SoXS.

### Photoluminescence characterizations

The steady-state PL spectra were carried out on an FLS980 spectrometer (Edinburgh Instruments). The excitation light with a wavelength of 550 nm was provided by a Xe lamp. The signals were recorded using a NIR charge-coupled-device (CCD) cooled to −80 °C with liquid nitrogen. TRPL measurements were carried out on an FLS980 spectrometer (Edinburgh Instruments). The excitation laser is a diode-pumped solid-state laser (485 nm, 20 MHz). The spot size is ~1.5 mm^2^. The laser intensity on the samples was tuned by a continuously variable neutral density filter wheel. The signals were eventually detected using a UV–Vis CCD thermoelectrically cooled to −20 °C.

### Reporting summary

Further information on research design is available in the Nature Research Reporting Summary linked to this article.

## Supplementary information

Supplementary Information

Solar Cells Reporting Summary

## Data Availability

The authors declare that the main data supporting the findings of this study are available within the article and its Supplementary Information files. Other data sets generated and/or analyzed during the current study are available from the corresponding authors upon reasonable request.
