# Supplementary material for: Pseudo-bilayer architecture enables high-performance organic solar cells with enhanced exciton diffusion length
Source: Nat Commun. 2021 Jan 20;12:468. doi: 10.1038/s41467-020-20791-z (PMC7817662; doi:10.1038/s41467-020-20791-z)
Supplement: Supplementary file 1 — Supplementary Information [file 41467_2020_20791_MOESM1_ESM.pdf]

Supplementary Information for

Pseudo-Bilayer Architecture Enables High-Performance Organic Solar Cells with Enhanced Exciton Diffusion Length

Kui Jiang[1,∇], Jie Zhang[2,∇], Zhengxing Peng[3], Francis Lin[2], Shengfan Wu[1], Zhen Li[2], Yuzhong Chen[4], He Yan[4★], Harald Ade[3★], Zonglong Zhu[1,2★] & Alex K.-Y. Jen[1,2★]

[1]Department of Materials Science and Engineering, City University of Hong Kong, Tat Chee Avenue, Kowloon 999077, Hong Kong

[2]Department of Chemistry, City University of Hong Kong, Tat Chee Avenue, Kowloon 999077, Hong Kong

[3]Department of Physics and Organic and Carbon Electronics Laboratory (ORaCEL), North Carolina State University, Raleigh, North Carolina 27695, USA

[4]Department of Chemistry and Energy Institute, The Hong Kong University of Science and Technology, Clear Water Bay, Kowloon 999077, Hong Kong

[∇]These authors contributed equally: Kui Jiang, Jie Zhang

[★]To whom correspondence should be addressed. Email: hyan@ust.hk; hwade@ncsu.edu; zonglzhu@cityu.edu.hk; alexjen@cityu.edu.hk

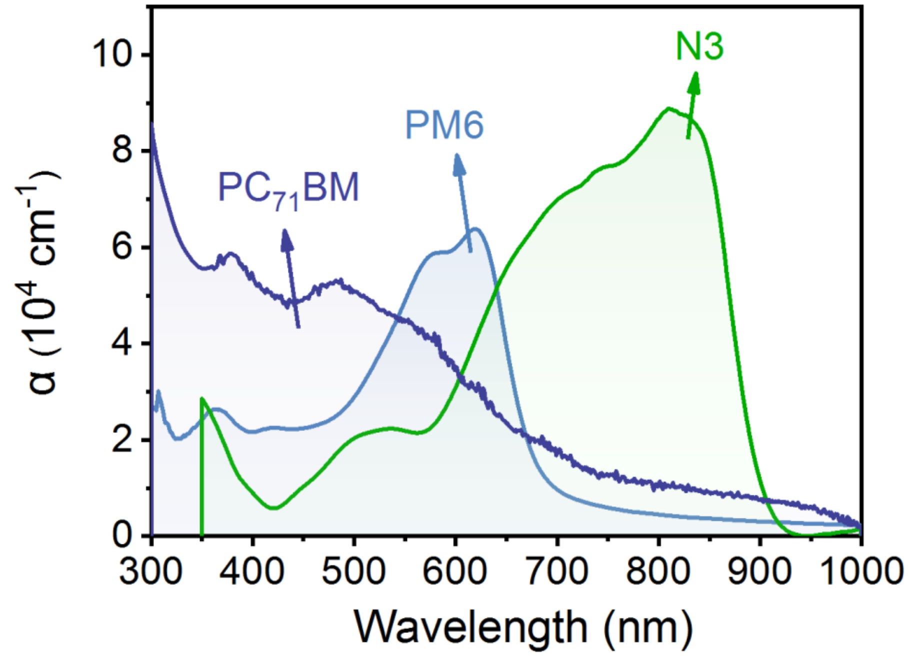

Supplementary Fig. 1 The absorption coefficient of the neat $PC_{71}BM$, PM6 and N3 films.

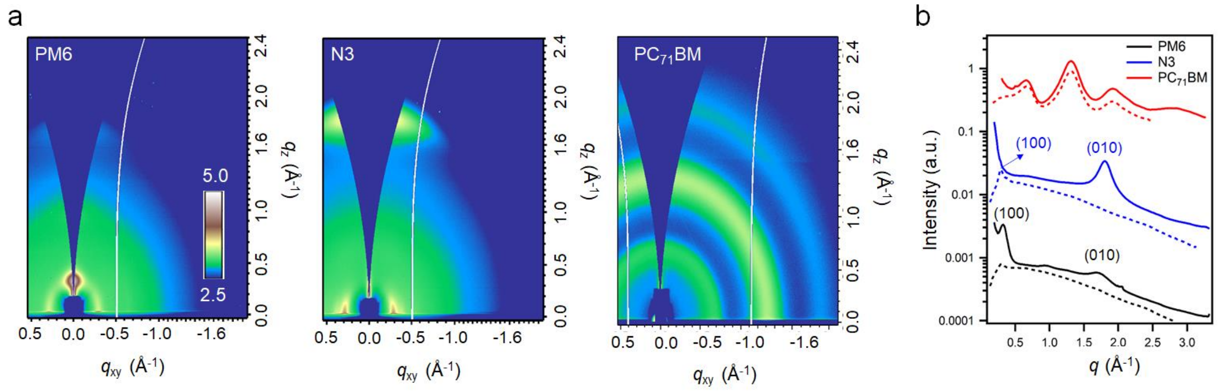

Supplementary Fig. 2 The 2D GIWAXS patterns (**a**) and the GIWAXS 1D profiles (**b**) of the neat $PC_{71}BM$, PM6 and N3 films.

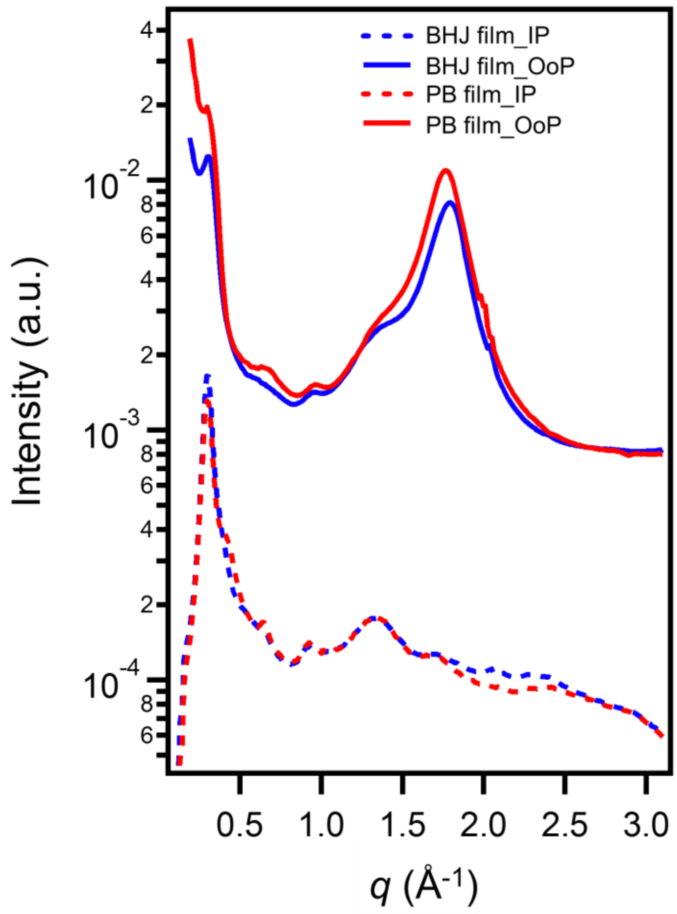

Supplementary Fig. 3 OoP and IP GIWAXS profiles of PM6:N3:$PC_{71}BM$ blend film with BHJ and PB architectures.

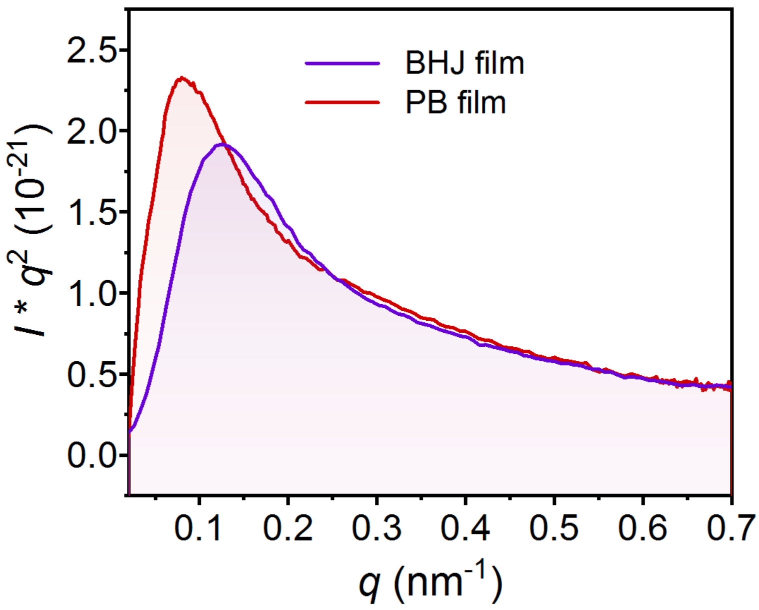

Supplementary Fig. 4 The resonant soft x-ray scattering (RSoXS) profiles of the PM6:N3:$PC_{71}BM$ blend films with BHJ and pseudo-bilayer structures.

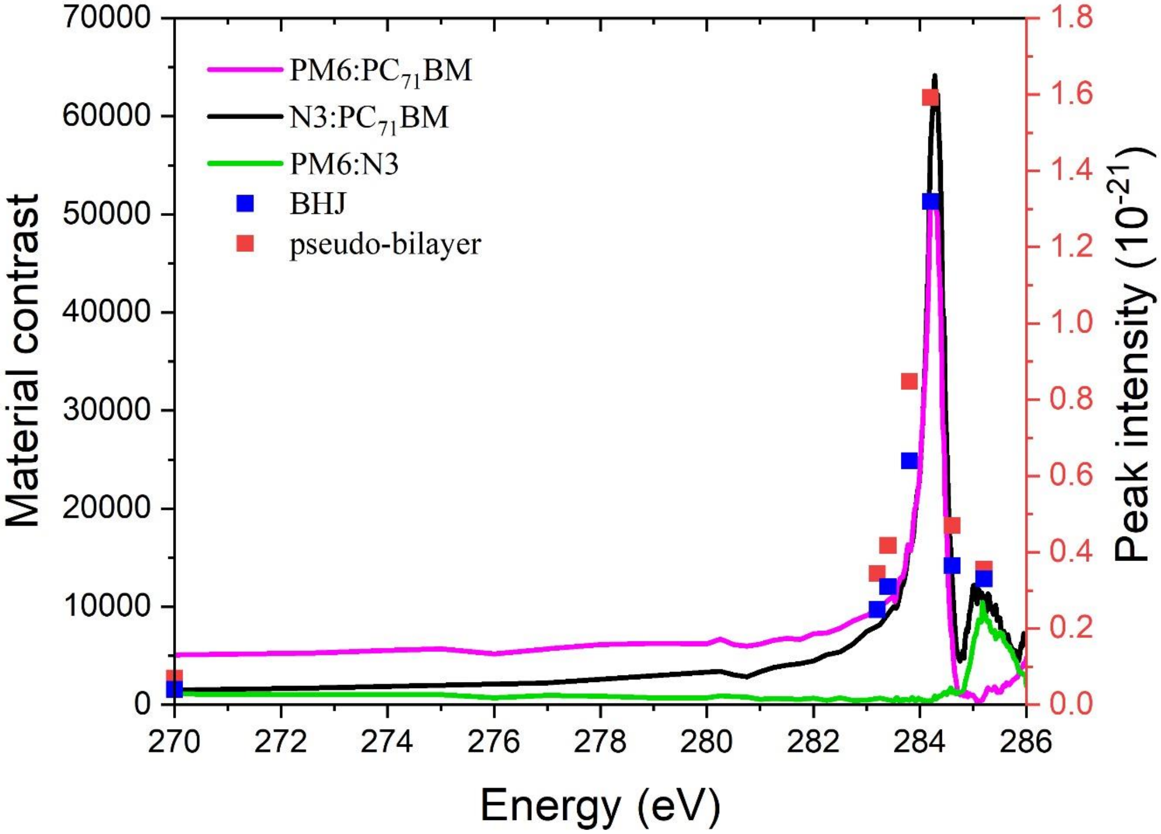

Supplementary Fig. 5 Comparison of measured relative scattering intensity against contrast between materials pairs. The measurements clearly show that the scattering is dominated by the $PC_{71}BM$ distribution against the low contrast PM6:N3 non-fullerene components.

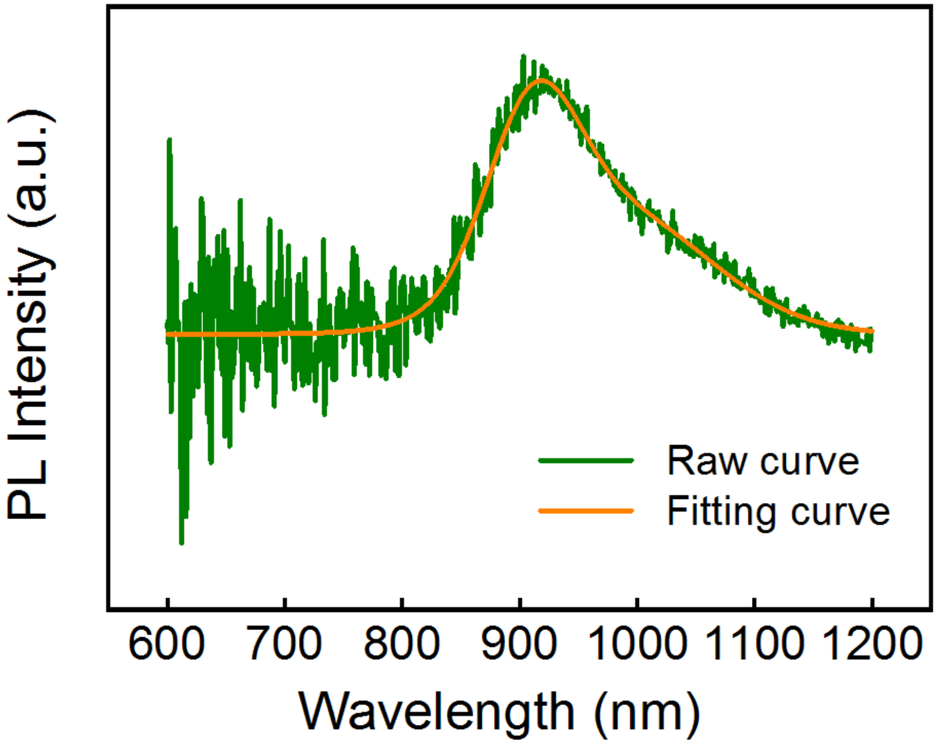

Supplementary Fig. 6 The raw PL signals recorded by our NIR detector (green curve) and the fitting curve acquired via multi-peak fitting method (orange curve).

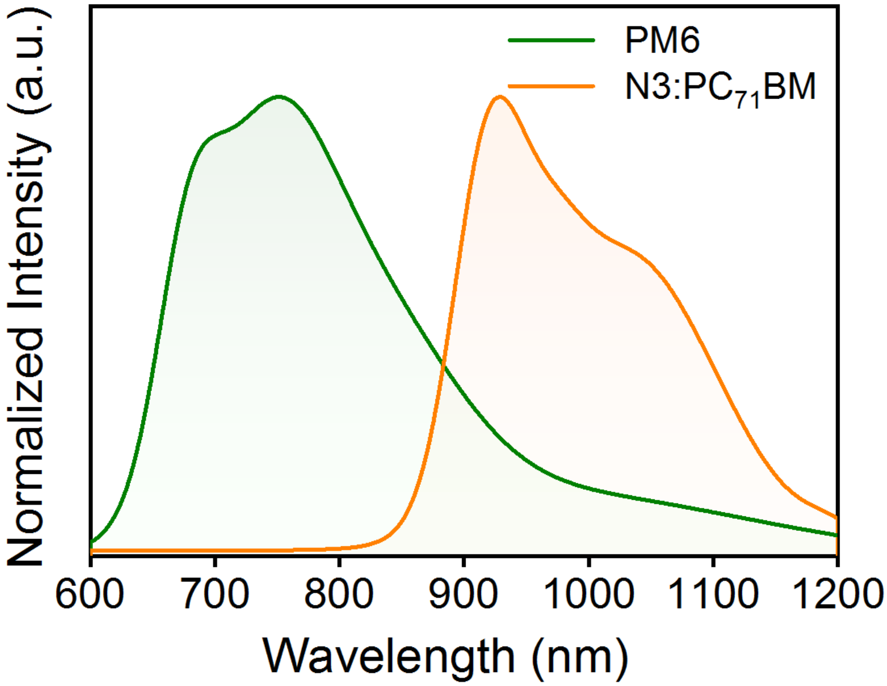

Supplementary Fig. 7 The PL spectra of neat PM6 and N3:PC71BM films.

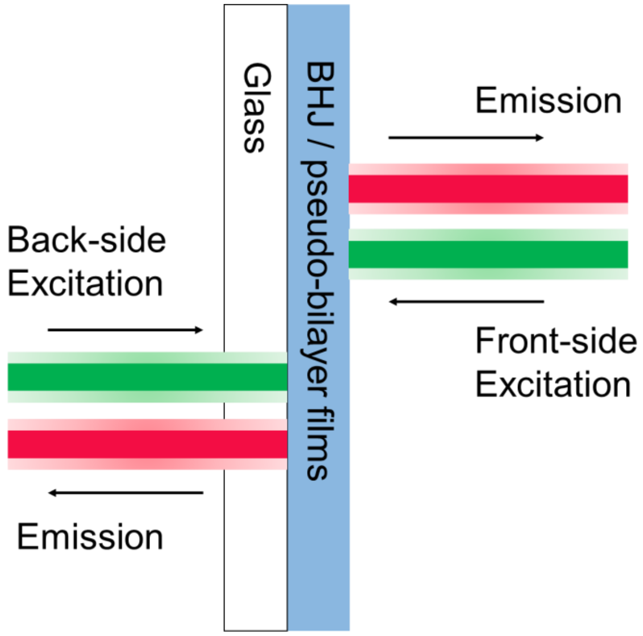

Supplementary Fig. 8 The schematic of excitation from the top and bottom film sides in the PL mapping experiments.

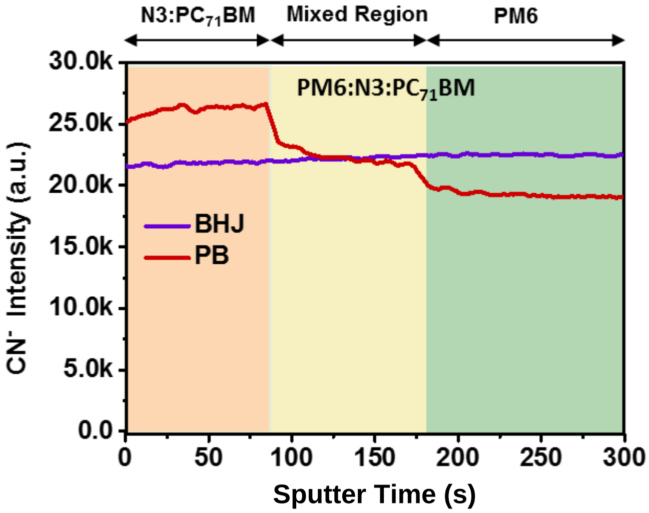

Supplementary Fig. 9 ToF-SIMS ion yield of BHJ and PB films is plotted as a function of sputtering time, which tracks the N3 molecule with the –CN^- group as the characteristic fragment.

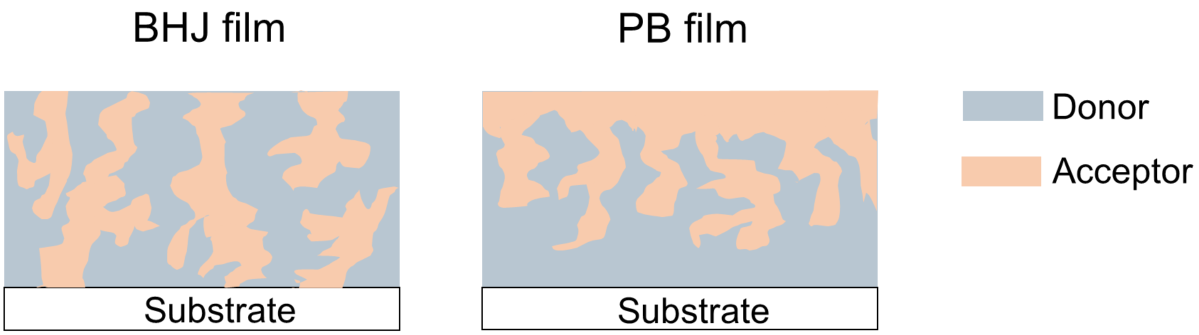

Supplementary Fig. 10 The schematic of the vertical morphology of films with BHJ and PB architectures.

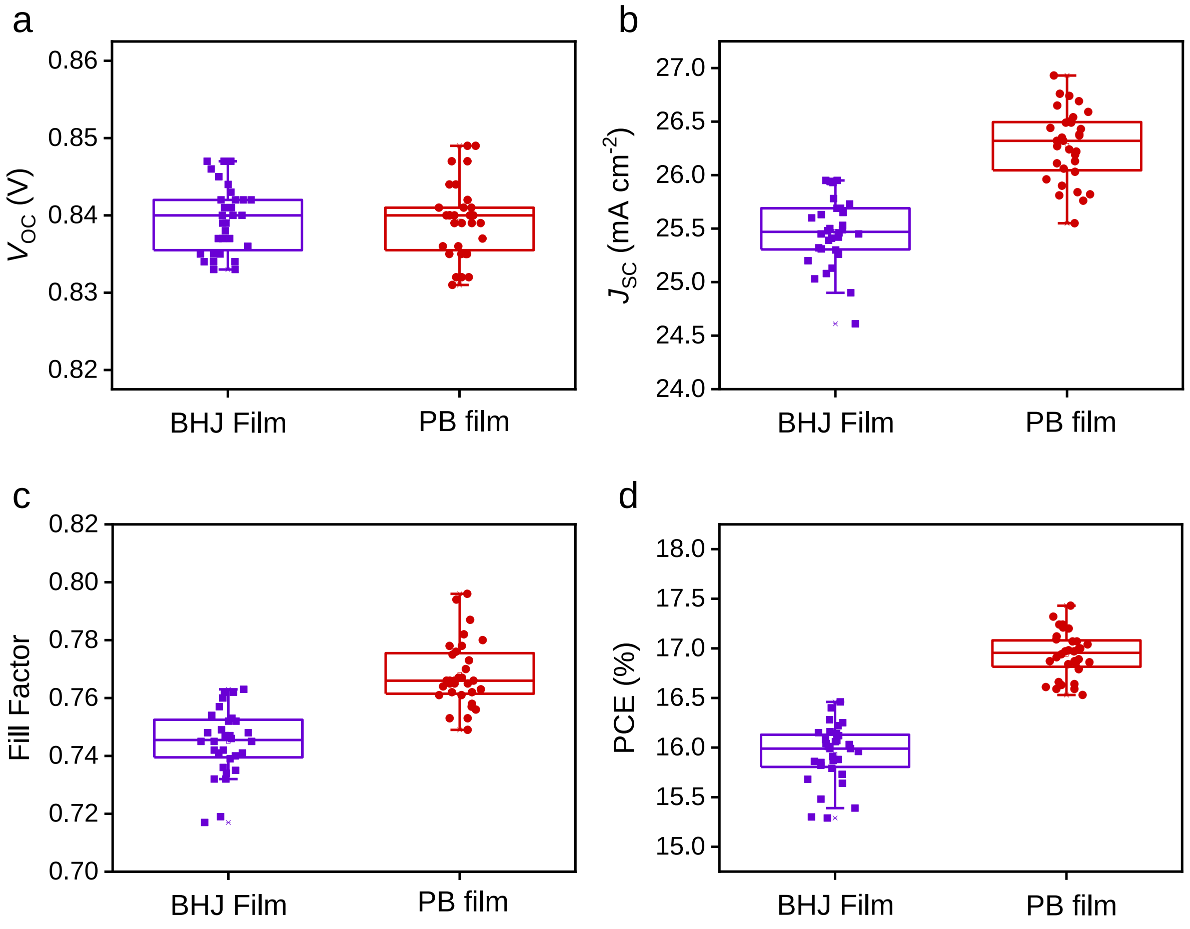

Supplementary Fig. 11 The performance statistic of the OSCs based on the BHJ and pseudo-bilayer films. These results were obtained from obtained from 30 devices with BHJ or PB architectures. The boxplots show the minimum, first quartile (Q_1 / 25th percentile), mean, third quartile (Q_3 / 75th percentile), and maximum for the photovoltaic performance of devices.

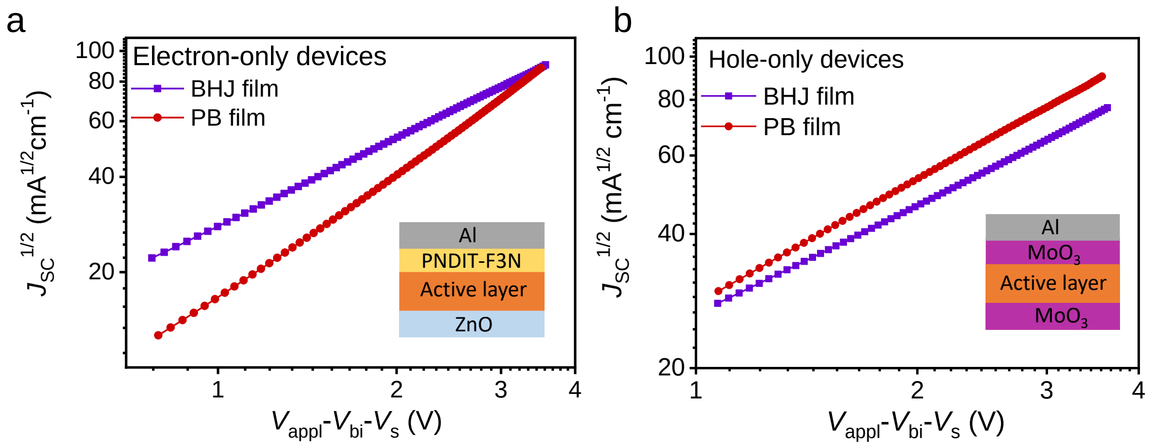

Supplementary Fig. 12 The current density-voltage (*J*-*V*) curves of the (**a**) electron- and (**b**) hole-only devices based on the BHJ and PB films. The insets are the structures of electron- and hole-only devices.

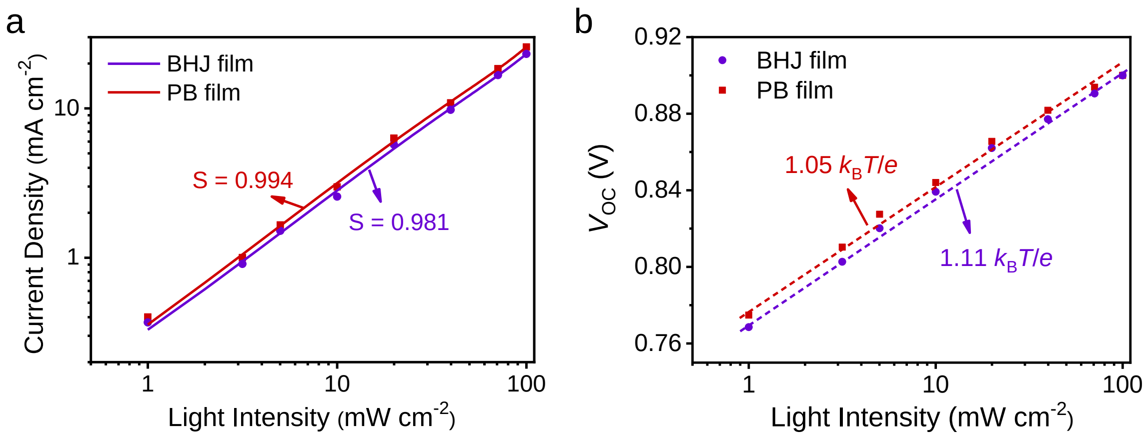

Supplementary Fig. 13 The light-dependent current density (**a**) and open-circuit voltage (**b**) of the BHJ and PB OSCs.

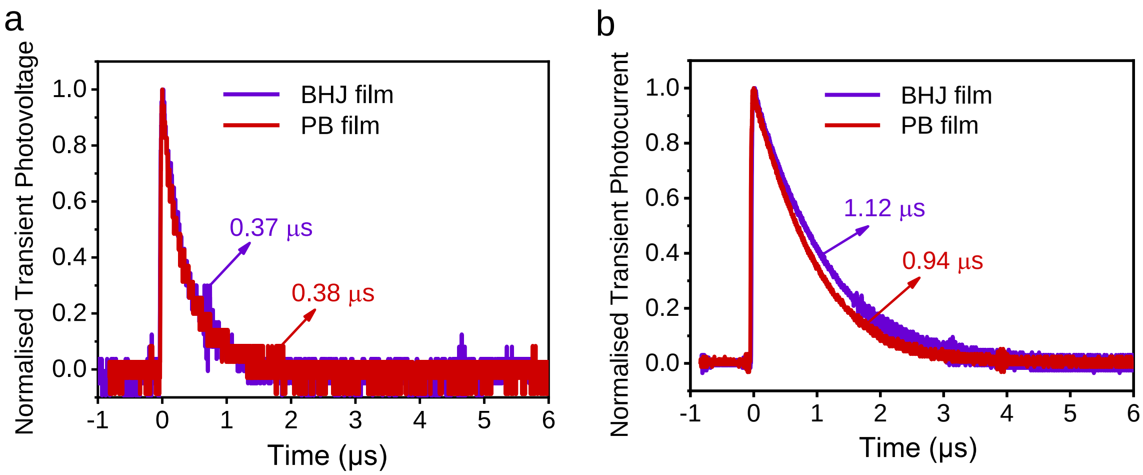

Supplementary Fig. 14 The transient photovoltage (**a**) and transient photocurrent (**b**) spectra of the devices based on BHJ and PB films.

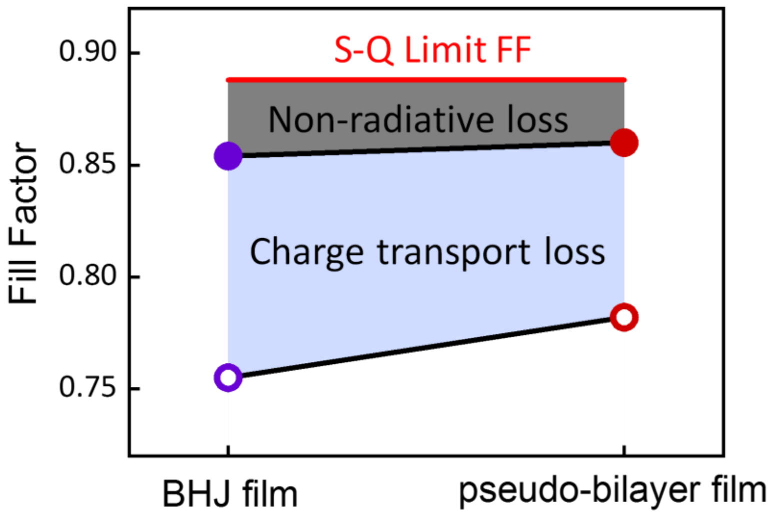

Supplementary Fig. 15 The FF loss analysis of the BHJ and PB OSCs.

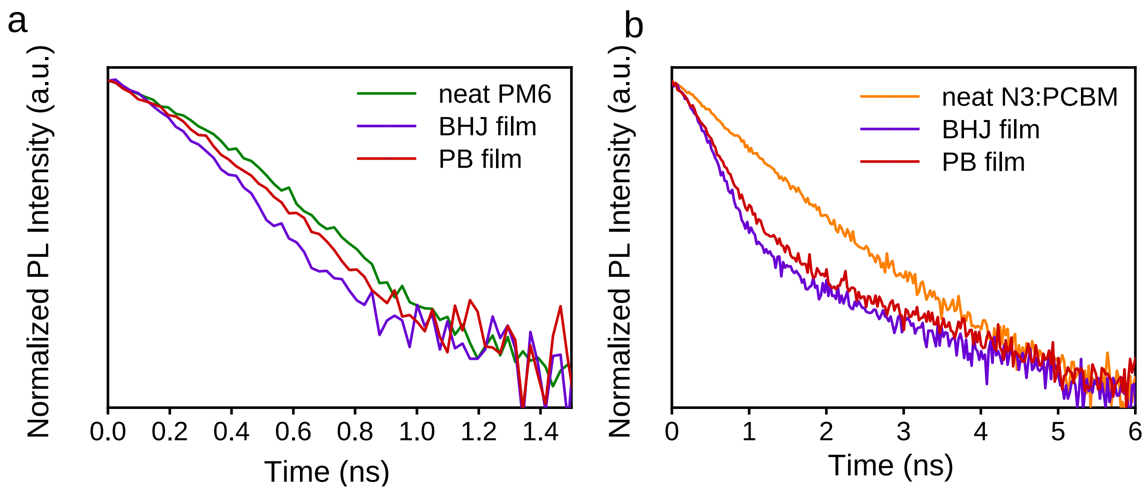

Supplementary Fig. 16 The PL decay plots of blend and neat films at 680 nm (**a**) and 840 nm (**b**), respectively. The neat films include the neat PM6 and neat N3 films. The blend films include the films with BHJ and PB architectures.

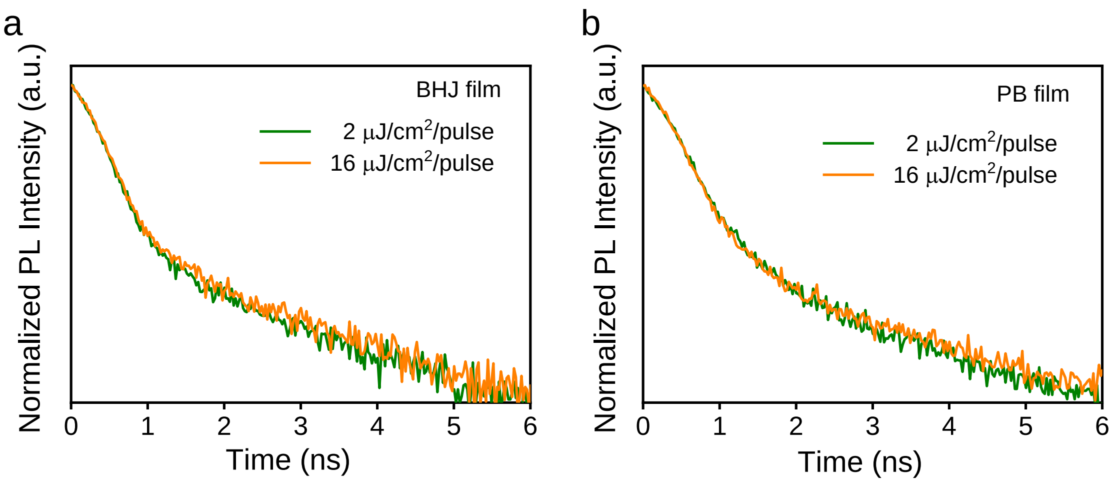

Supplementary Fig. 17 The PL decay plots of the BHJ (**a**) and PB (**b**) films under the excitation laser with low and high power density.

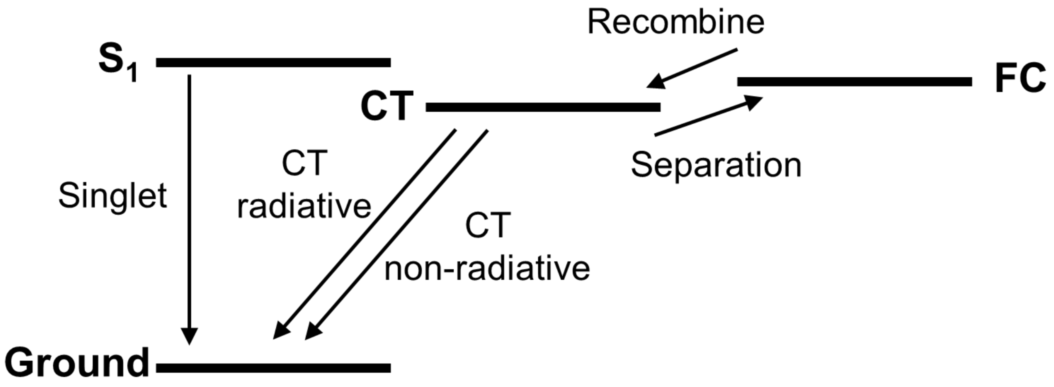

Supplementary Fig. 18 The schematic of the transfer between singlet, charge transfer (CT) state and charge separation. S_1: singlet state. CT: charge transfer. FC: free charge.

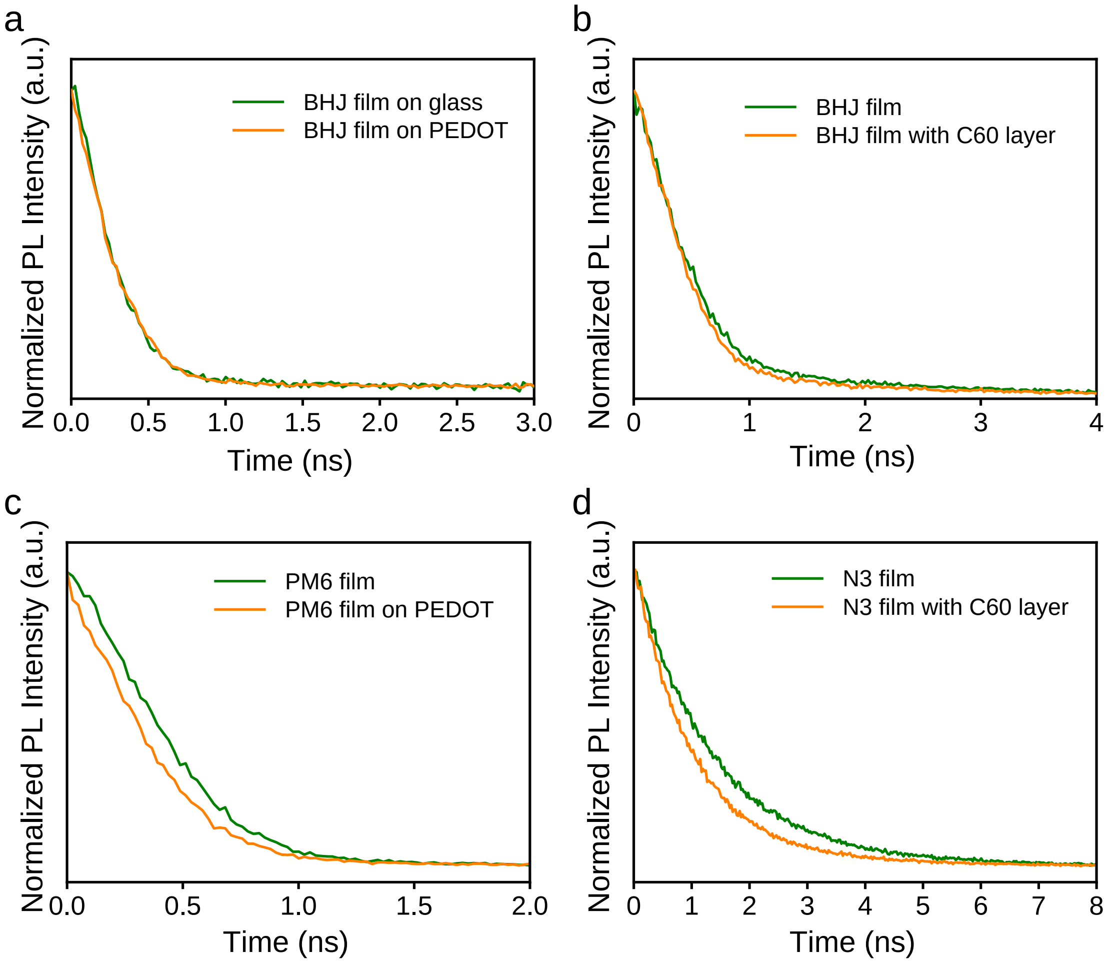

Supplementary Fig. 19 The PL decay plots of the BHJ film at (**a**) 680 nm and (**b**) 840 nm. The PL decay plots of (**c**) the PM6 film at 680 nm and (**d**) N3 film at 840 nm.

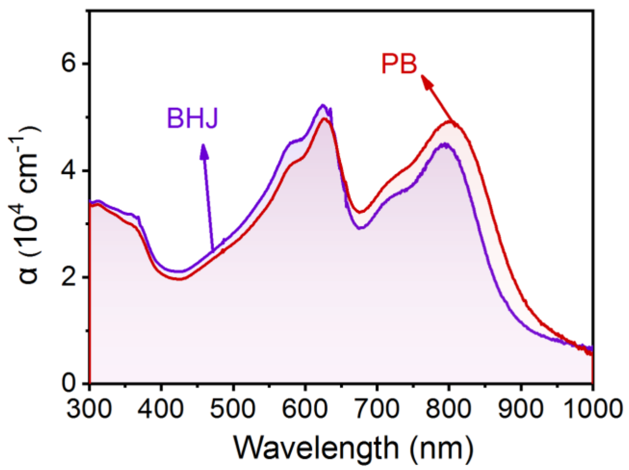

Supplementary Fig. 20 The absorption coefficient spectra of PM6:N3:$PC_{71}BM$ blend film with BHJ and PB architectures.

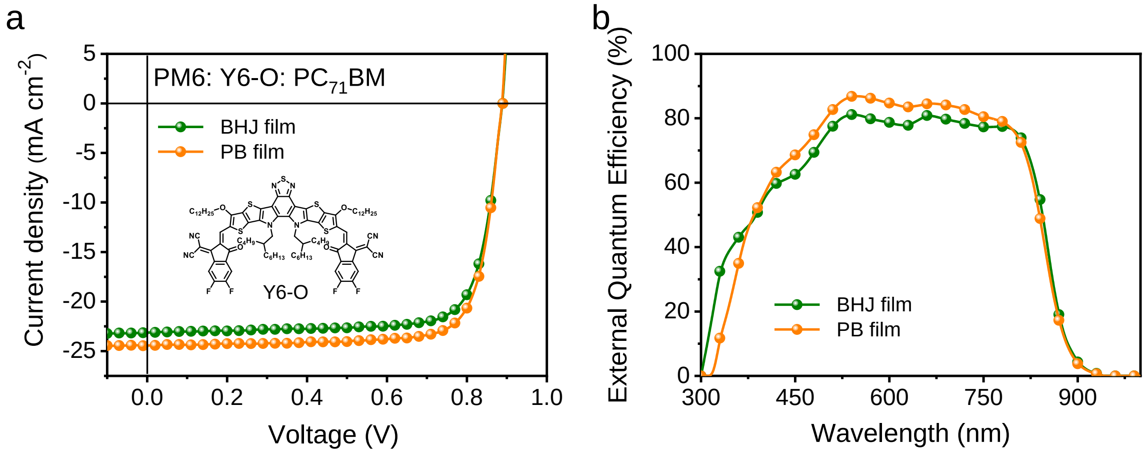

Supplementary Fig. 21 The current density-voltage (*J*-*V*) and external quantum efficiency curves for the devices based on the PM6:Y6-O:$PC_{71}BM$ blend films with BHJ and pseudo-bilayer structures.

OPV 1-J Cell

Device Temperature: 25.0 ± 1.5 °C
Device Area: 0.04059 cm^2 ± 0.83%
Spectrum: ASTM G173 global
Irradiance: 1000.0 W/m^2

NREL
NATIONAL RENEWABLE ENERGY LABORATORY
X25 IV System
PV Performance Characterization Team

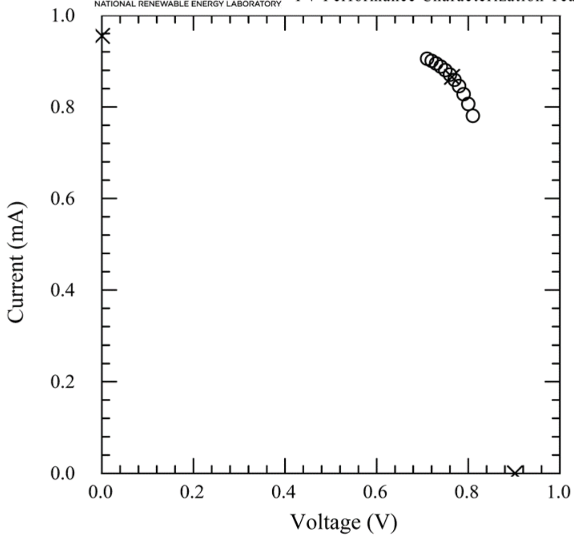

V_{oc} = 0.902 V ± 1.36%
I_{sc} = 0.955 mA ± 0.71%
J_{sc} = 23.53 mA/cm^2 ± 1.43%
Fill Factor = (76.84 ± 1.16)%

I_{max} = 0.866 mA ± 0.76%
V_{max} = 0.765 V ± 1.46%
P_{max} = 0.662 mW ± 1.32%
Efficiency = (16.31 ± 0.27)%

Asymptotic P_{max} scan

Sweep time: 1541 s

Supplementary Fig. 22 Independent efficiency certification of OSCs based on the PM6:Y6-O:$PC_{71}BM$ blend film with pseudo-bilayer structure by an accredited institute of National Renewable Energy Laboratory (NREL, USA), giving a PCE of 16.31% (J_{SC} of 23.53 mA cm^{-2}, V_{OC} of 0.902 and FF of 76.84%), which is among the highest values for the certified efficiency for ternary OSCs. This certified PCE was obtained under the new stress-test certification protocol.

Supplementary Table 1. The parameters of GIWAXS 1D profiles for the PM6:N3:$PC_{71}BM$ films with BHJ and PB architectures.

	BHJ film						PB film				
$\boldsymbol{q_z}$ (Å^{-1})	d (Å^{-1})	FWHM (Å^{-1})	L_C (Å^{-1})	g	I_{norm}	$\boldsymbol{q_z}$ (Å^{-1})	d (Å^{-1})	FWHM (Å^{-1})	L_C (Å^{-1})	g	I_{norm}
1.71	3.7	0.534	12	22%	0.62	1.73	3.6	0.46	14	21%	1
1.79	3.5	0.185	34	13%	0.38	1.77	3.5	0.17	37	12%	0.44

Supplementary Table 2. The parameters of resonant soft x-ray scattering (RSoXS) profiles for the PM6:N3:$PC_{71}BM$ films with BHJ and PB architectures.

Film Architectures	Peak position (nm^{-1})	Long period (nm)	Average Domain Size (nm)	Root-mean-square composition variation
BHJ film	0.325	53	26.5	0.94
PB film	0.303	76	38	1

Supplementary Table 3. The statistic of the performance parameters for the devices based on the PM6:N3:$PC_{71}BM$ films with BHJ and PB architectures. These results show the mean value and standard deviation.

**Film Architectures**	V_{OC} **(V)**	J_{SC} **(mA cm^{-2})**	**FF**	**PCE (%)**
BHJ film	0.839 ± 0.004	25.47 ± 0.32	0.745 ± 0.011	15.93 ± 0.29
PB film	0.839 ± 0.005	26.28 ± 0.33	0.768 ± 0.011	16.94 ± 0.23

Supplementary Table 4. The mobilities of electrons and holes in the PM6:N3:$PC_{71}BM$ films with BHJ and PB architectures.

**Film Architectures**	μ_e ($cm^2\ V^{-1}\ S^{-1}$)	μ_h ($cm^2\ V^{-1}\ S^{-1}$)	μ_h / μ_e
BHJ film	4.6×10^{-4}	6.0×10^{-4}	1.30
PB film	5.7×10^{-4}	7.1×10^{-4}	1.24

Supplementary Table 5. The average PL lifetime in the PM6:N3:$PC_{71}BM$ films with BHJ and PB architectures at 680 nm and 840 nm, respectively. The PL decay at 680 nm and 840 nm were well fitted to exponential and bi-exponential functions, respectively.

**PL Signals**	**Film Architectures**	$\boldsymbol{A_1}$ (%)	$\boldsymbol{\tau_1}$ (ns)	$\boldsymbol{A_2}$ (%)	$\boldsymbol{\tau_2}$ (ns)	$\boldsymbol{\tau_{avg}}$ (ns)
at 680 nm	PM6 film	100	0.29	---	---	0.29
	BHJ film	100	0.22	---	---	0.22
	PB film	100	0.26	---	---	0.26
at 840 nm	N3 film	94.2	1.31	5.8	3.69	1.45
	BHJ film	93.3	0.38	6.7	3.81	0.61
	PB film	93.4	0.44	6.6	3.76	0.66

Supplementary Table 6. The parameters for the calculation of exciton diffusion length in the PM6:N3:$PC_{71}BM$ films with BHJ and PB architectures.

	**Film Architectures**	**PL quenching**	**Thickness** (nm)	**Absorption Coefficient** (cm^{-1})	**Exciton Diffusion Length** (nm)
in PM6 domains	PM6 film	0.078	104	3310.816	8.22
	BHJ film	---	94	2661.300	---
	PB film	0.071	99	2513.647	7.14
in N3 domains	N3 film	0.194	101	1564.553	19.68
	BHJ film	0.079	94	2661.300	7.53
	PB film	0.171	99	2513.647	17.07

Supplementary Table 7. The performance parameters of the champion devices based on the PM6:Y6-O:$PC_{71}BM$ films with BHJ and PB architectures. The values in the brackets are the J_{SC} calculated from the EQE.

Film Architectures	V_{OC} (V)	J_{SC} (mA cm^{-2})	FF	PCE (%)
BHJ film (PM6:Y6-O:$PC_{71}BM$)	0.900	23.30 (22.60)	0.770	16.14
PB film (PM6:Y6-O:$PC_{71}BM$)	0.900	24.30 (23.90)	0.790	17.27
PB film, certified (PM6:Y6-O:$PC_{71}BM$)	0.902	23.53	0.768	16.31

Supplementary Note 1

In this work, the PL spectra were employed to distinguish the PL signals originated from donor or acceptor domains in the blend films. Comparing the PL spectra of blend films to that of neat films (Fig. 2c, d and Supplementary Fig. 6), the PL signals in the range between 600 nm and 800 nm should be contributed by the singlet excitons in PM6 domains, while the PL signals of a wavelength larger than 800 nm would be dominated by N3 domains. Therefore, the PL signals at 680 nm and 840 nm were employed to study the exciton behaviors in PM6 and N3 domains in the blend films, respectively.

Supplementary Note 2

In this work, we employed the PL quenching method, which established by Markov *et al.*, to estimate the exciton diffusion length in our films.[1] In general, this method was used to calculate the exciton diffusion length in neat film. For the films with BHJ and PB architectures, the reasons for using this method are that: (1) the PL signals obtained from these films only originated from the geminate recombination, such as exciton or CT states recombination. Because the CT states only existed at the D/A interface, the PL quenching just described the exciton dynamic behaviors in the films without the interference from high-order recombination; (2) the film with BHJ architecture is a well-blended film, which could be regarded as a "neat film" composed of mixed D/A domains. Considering the film thickness of 99 nm and the reasonable exciton diffusion length in organic materials (~ 20 nm), the high J_{SC} implies that the most region in the film with PB architecture should be well-blended. Therefore, we treated the film with BHJ or PB architecture as one "black-box" and used the output PL signals of the "black-box" to acquire the statistic information of the exciton dynamic behaviors in the film.

The PL quenching efficiency, Q, is obtained with the time-integrated approach:[1]

$$Q = 1 - \frac{\int \mathrm{TRPL_{quench}}\, dt}{\int \mathrm{TRPL_{reference}}\, dt}$$

where the $\mathrm{TRPL_{quench}}$ and $\mathrm{TRPL_{reference}}$ are the PL decay for the film with and without quenching layer. Markov *et al*. reported that Q can be described as a function of film thickness (L):

$$Q = \frac{(\alpha^2 L_\mathrm{D}^2 + \alpha L_\mathrm{D} \tanh(L/L_\mathrm{D}))\exp(-\alpha L) - \alpha^2 L_\mathrm{D}^2 (\cosh(L/L_\mathrm{D}))^{-1}}{(1-\alpha^2 L_\mathrm{D}^2)\big(1-\exp(-\alpha L)\big)}$$

where α is the absorption coefficient. After measuring α, L and Q, L_D will be the only unknown parameter in this equation and can be extracted by the numerical calculation. In this work, the $\mathrm{TRPL_{reference}}$ was the PL decay in the films prepared on glass.

In addition, the films with PB structure were possessed with the neat PM6 layer, mixed layer and neat N3:$PC_{71}BM$ layer from bottom to top. The exciton behaviors in donor and acceptor domains

cannot be simultaneously studied with a PB film deposited on a PL quenching layer. Therefore, we used the PB film prepared on PEDOT:PSS layer to study the exciton behaviors in donor domains, while the exciton behaviors in acceptor domains were studied through the PB film coated with a C60 layer. In order to properly compare the exciton behavior in BHJ and PB film, the sample architecture of PEDOT:PSS/PB (or BHJ) film and PB (or BHJ) film/C60 were utilized to study the exciton behaviors in donor domains (Fig. 4a and Supplementary Fig. 19a) and acceptor domains (Fig. 4b and Supplementary Fig. 19b), respectively.

References

1. Markov, D. E., Amsterdam, E., Blom, P. W. M., Sieval, A. B. and Hummelen, J. C. Accurate measurement of the exciton diffusion length in a conjugated polymer using a heterostructure with a side-chain cross-linked fullerene layer. *J. Phys. Chem. A.* **109**, 5266-5274 (2005).
