# Supplementary material for: Pseudo-bilayer architecture enables high-performance organic solar cells with enhanced exciton diffusion length
Source: Nat Commun. 2021 Jan 20;12:468. doi: 10.1038/s41467-020-20791-z (PMC7817662; doi:10.1038/s41467-020-20791-z)
Supplement: Supplementary file 2 — Solar Cells Reporting Summary [file 41467_2020_20791_MOESM2_ESM.pdf]

nature research

Corresponding author(s): He Yan, Zonglong Zhu and Alex K.-Y. Jen

Solar Cells Reporting Summary

Nature Research wishes to improve the reproducibility of the work that we publish. This form is intended for publication with all accepted papers reporting the characterization of photovoltaic devices and provides structure for consistency and transparency in reporting. Some list items might not apply to an individual manuscript, but all fields must be completed for clarity.

For further information on Nature Research policies, including our data availability policy, see Authors & Referees.

ü Experimental design

Please check: are the following details reported in the manuscript?

1. Dimensions

Area of the tested solar cells — [x] Yes [ ] No
The active area of the conventional solar cell fabricated in our lab is 7mm2.

Method used to determine the device area — [x] Yes [ ] No
The electronic active area of the cell, which was 7mm2, is defined by the overlap of the ITO electrode and metal electrode. When we make the measurement of devices, a metal mask with 5.9mm2 is used to define the device active area via an aperture aligned with the device area.

2. Current-voltage characterization

Current density-voltage (J-V) plots in both forward and backward direction — [ ] Yes [x] No
In general, organic solar cells show the same current density-voltage curves in both forward and backward direction. Therefore, we only scan the solar cells in forward direction.

Voltage scan conditions
For instance: scan direction, speed, dwell times — [x] Yes [ ] No
We scan the solar cells in the range of -0.2~1v with 0.6 V/s.

Test environment
For instance: characterization temperature, in air or in glove box — [x] Yes [ ] No
The measurements were performed in air at temperature at room temperature.

Protocol for preconditioning of the device before its characterization — [ ] Yes [x] No
No preconditioning protocol was applied.

Stability of the J-V characteristic
Verified with time evolution of the maximum power point or with the photocurrent at maximum power point; see ref. 7 for details. — [x] Yes [ ] No
NREL provided us with the stability certification of J-V characteristic at maximum power point in this work. Details are given in Supplementary Fig. 22.

3. Hysteresis or any other unusual behaviour

Description of the unusual behaviour observed during the characterization — [ ] Yes [x] No
There is no unusual behaviour observed during the characterization, i.e, hysteresis.

Related experimental data — [ ] Yes [x] No
We didn't find the unusual behaviour.

4. Efficiency

External quantum efficiency (EQE) or incident photons to current efficiency (IPCE) — [x] Yes [ ] No
See the Fig. 3c and Supplementary Fig. 21. For the EQE measurement, We show details in the Methods section.

A comparison between the integrated response under the standard reference spectrum and the response measure under the simulator — [x] Yes [ ] No
The difference between the integrated current from EQE and the short-circuit current from J-V curve measured under AM 1.5G solar simulator is within 3% difference which is within the accuracy confidence of the measurements.

For tandem solar cells, the bias illumination and bias voltage used for each subcell — [ ] Yes [x] No
We did not make the tandem solar cells in this work.

5. Calibration

Light source and reference cell or sensor used for the characterization — [x] Yes [ ] No
A Newport simulator was used as light source and the light intensity was calibrated with a standard single-crystal Si solar cell made by PV Measurement. Details were shown in the Methods section.

Confirmation that the reference cell was calibrated and certified

- [x] Yes
- [ ] No

A standard single-crystal Si solar cell as the reference cell was purchased from PV Measurement and calibrated by Newport. We show details in the Method section.

Calculation of spectral mismatch between the reference cell and the devices under test

- [ ] Yes
- [x] No

We didn't calculated the spectral mismatch between the reference cell and devices. But one of our best cells was sent to NREL for certification.

6. Mask/aperture

Size of the mask/aperture used during testing

- [x] Yes
- [ ] No

Two mask sizes were used during testing with 0.04 cm2 or 0.059 cm2

Variation of the measured short-circuit current density with the mask/aperture area

- [ ] Yes
- [x] No

There is no obvious variation in the measured short-circuit current density with masks.

7. Performance certification

Identity of the independent certification laboratory that confirmed the photovoltaic performance

- [x] Yes
- [ ] No

the National Renewable Energy Laboratory (NREL)

A copy of any certificate(s)
Provide in Supplementary Information

- [x] Yes
- [ ] No

We provided the certification in Supplementary Fig. 22

8. Statistics

Number of solar cells tested

- [x] Yes
- [ ] No

We have tested over 30 cells in our lab.

Statistical analysis of the device performance

- [x] Yes
- [ ] No

We have given statistical data of device performance in Fig. 3d and Supplementary Fig. 11

9. Long-term stability analysis

Type of analysis, bias conditions and environmental conditions
For instance: illumination type, temperature, atmosphere humidity, encapsulation method, preconditioning temperature

- [ ] Yes
- [x] No

We didn't make the long-term stability. Because our work is mainly focus on understanding the detail process of the exciton generation and diffusion process based on the different processing method. The stability is not very important.
